# Agony of Choice: Caudal Block versus Ilioinguinal/Iliohypogastric Nerve Block in Unilateral Orchidopexy

**DOI:** 10.3390/children11070800

**Published:** 2024-06-29

**Authors:** Aybike Hofmann, Bernhard Koller, Franziska Vauth, Pirmin I. Zöhrer, Gregor Badelt, Wolfgang H. Rösch

**Affiliations:** 1 Clinic St. Hedwig, Department of Paediatric Urology, University Medical Center Regensburg, 93049 Regensburg, Germany; franziska.vauth@barmherzige-regensburg.de (F.V.); wolfgang.roesch@barmherzige-regensburg.de (W.H.R.); 2 Clinic St. Hedwig, Department of Paediatric Anaesthesia, 93049 Regensburg, Germany

**Keywords:** inguinal surgery, regional anesthesia, ultrasound guided, intra-/postoperative pain, ambulant surgery

## Abstract

**Objective:** This prospective study aimed to compare the efficacy of caudal block (CB) and ilioinguinal/iliohypogastric nerve block (IINB) for providing additional analgesia during unilateral orchidopexy. **Methods:** Seventy-one boys aged <48 months, classified as ASA I/II, were assigned into CB (*n* = 37) and IINB (*n* = 34) groups. Outcome measures included intra- and postoperative analgesic requirements, pain scores, and administration duration. Additional intraoperative analgesia was administered for a 10% increase in heart rate, while postoperative pain was assessed using the Children’s and Infants Postoperative Pain Scale (CHIPPS), with scores >4 prompting supplementary analgesia. Monitoring was extended for 24 h post-surgery. **Results:** CB significantly reduced the need for intraoperative (*p* < 0.001) and early postoperative (*p* = 0.008) analgesia compared to IINB. However, the CB group exhibited a slightly higher but non-significant analgesic requirement on the ward. No clinically relevant side effects were observed in either group. **Conclusions:** Both CB and IINB are effective and safe methods for providing regional analgesia during orchidopexy. CB demonstrates superior efficacy intraoperatively and in the early postoperative period, while IINB may offer advantages in the later recovery phase. However, additional analgesia is often required for orchidopexy, especially in outpatient settings.

## 1. Introduction

Cryptorchidism is a common congenital anomaly in boys, affecting approximately 3% of male newborns [1]. In almost 50% of cases, the undescended testis (UDT) descends spontaneously within the first year of life [2]. Despite this high rate of spontaneous descent, orchidopexy remains one of the most frequently performed surgical interventions in children in Germany, with 12,000–18,000 procedures conducted annually [3].

Orchidopexy is considered one of the most painful inguinal procedures due to the traction exerted on the testicle and the spermatic cord [4]. Therefore, ensuring adequate analgesia is crucial, especially in outpatient surgery, to prevent unnecessary hospital readmissions.

Hence, in recent years, a combination of general anesthesia along with either a caudal block (CB) or ilioinguinal/iliohypogastric block (IINB) as regional anesthesia has gained acceptance for inguinal procedures.

In the late 1980s, Markham and Hannallah concluded that the safety and effectiveness of both CB and IINB are comparable for postoperative pain relief, despite their specific advantages and disadvantages [5,6]. Subsequent studies have also compared these regional techniques and often found them to be equivalent. However, it is important to note that in many of these studies, interventions other than orchidopexy were included in the assessment of inguinal procedures. In addition, the assessments primarily focused on the immediate postoperative outcome and did not consider the intraoperative course or the 24 h course, which are crucial aspects of a potentially outpatient procedure [7,8,9].

The aim of this study was to evaluate and compare the effectiveness of CB and IINB in orchidopexy, both intraoperatively and postoperatively. This is particularly relevant given the increasing trend of performing this procedure on an outpatient basis.

## 2. Materials and Methods

### 2.1. Study Design and Population

**Figure 1:**
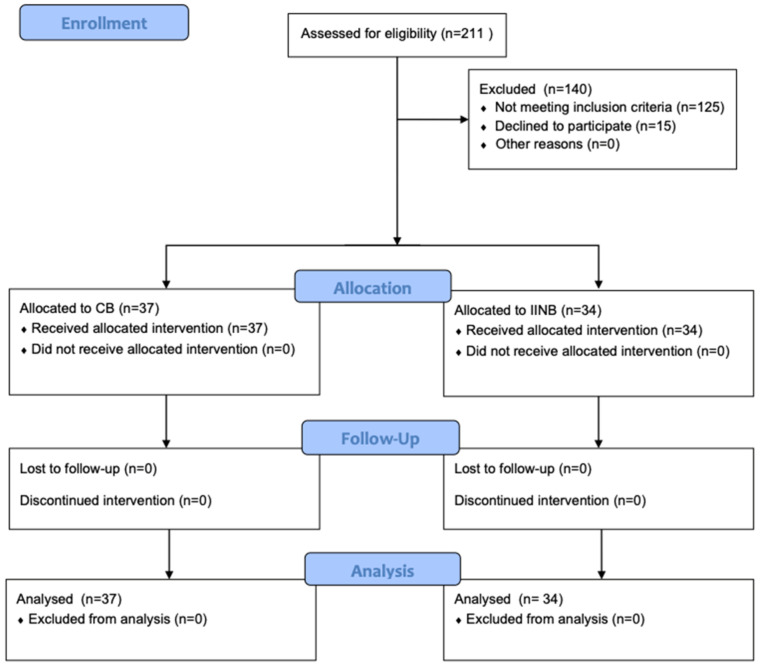
CONSORT flow diagram of participants’ disposition throughout study.

This prospective and explorative trial included 71 boys aged between 9 months and 4 years with ASA (American Society of Anaesthesiology) physical status I and II who underwent elective unilateral orchidopexy at our department (a full member of ERN eUROGEN) in a one-day inpatient setting. Parental consent was obtained after explaining the procedure. Exclusion criteria for the study included bilateral orchidopexy, re-orchidopexy, intolerance or allergic reaction to any product used in the study, and inability to provide written informed consent. In order to obtain a valid effect estimate, a minimum of 30 patients were included in each group [10]. The CONSORT flow diagram below outlines the detailed progression of participants through each phase of our trial, including enrollment, intervention allocation, follow-up, and data analysis. (see Figure 1)

The study protocol was approved by the Institutional Ethics Committee of the University of Regensburg (No. 16-101-0115).

After obtaining written consent from the parents, the preoperative anesthesiologist suggested the type of regional anesthesia (RA). The patients were then divided into two groups based on the type of RA used: CB group and IINB group.

The primary outcome was the intraoperative and postoperative opioid in the PACU and NSAID requirements in the ward. The secondary outcome parameters were the time until the necessity for additional pain medication and the pain score at that time. The tertiary outcome parameters were the occurrence of side effects.

### 2.2. Study Intervention

General anesthesia was induced in all patients before performing RA. RA was typically performed under sterile conditions and with ultrasound guidance (LOGIQ™℮, GE Healthcare, Wauwatosa, WI, USA). For patients weighing less than 10 kg, a hockey stick probe (4–15 MHz) was used, while those weighing over 10 kg underwent ultrasound with a linear probe (4.2–13 MHz). Patients in the CB group were positioned in the left lateral position with their knees and hips flexed at 90 degrees. This position causes a cranial shift of the dural sheath, minimizing the risk of dural perforation. The sacral hiatus was accessed using a 22-gauge caudal needle (Epican^®^ Paed caudal needle, 45° Crawford type bevel for epidural anesthesia/analgesia, B. Braun^®^ Medical Inc., Melsungen, Germany) inserted at a 45–60° angle to the skin level. Once the ligamentum sacrococcygeum was passed, the needle was lowered to a 20–30° angle and advanced 3–5 mm. An adrenaline test dose (1:200,000 ≙ 5 μg/mL) was then administered. Following a negative test with no pulse rise or T-wave elevation, 1.2 mL/kg bw of 0.2% ropivacaine [11] with 2 μg/kg bw of clonidine was administered over 1 min, with repeated aspiration, ECG monitoring, and continuous sonographic demonstration of the ventral dura shift with cranial dissemination.

Patients in the IINB group were maintained in a supine position. The Musculus obliquus externus and internus and the Musculus transversus abdominis were then identified via ultrasound imaging. Between the latter two muscles, the nervus ilioinguinal and the nervus iliohypogastricus were identified. A 22-gauge Ultraplex^®^ 360 needle (30°, 0.7 × 50 mm, B. Braun^®^ Medical Inc., Melsungen, Germany) was inserted parallel to the ultrasound probe from the median to the anterior iliac spine. After confirming the target nerve and a negative aspiration test, 0.2 mL/kg body weight of 0.2% ropivacaine was administered. The correct administration was confirmed by sonographic visualization of the internal oblique muscle and transversus abdominis muscle drifting. All blocks were performed by an experienced anesthesiologist, mainly by one of the authors (BK).

### 2.3. Preoperative Management

Depending on the patient’s age, preoperative fasting times were 4 h for milk, 6 h for solid food, and 1 h for clear fluids. All patients received oral premedication of Midazolam at a dose of 0.5 mg/kg bw. An EMLA dressing was applied to both antecubital veins in all patients, and an IV catheter was inserted.

### 2.4. Anesthetic and Perioperative Management

The anesthetic management was standardized for all patients. Standard monitoring techniques were employed. Adequate perioperative hydration was achieved by administering a balanced full electrolyte solution or a glucose 1% infusion at a rate of 10 mL/kgbw/h via an infusion pump. Anesthesia was induced via an IV line using propofol at a dose of 3–5 mg/kg body weight and fentanyl at a dose of 2 μg/kg body weight. Alternatively, in cases where an IV line was difficult to place, anesthetic induction was performed using inhalation of sevoflurane at a concentration of 6–8 Vol% in a nitrous oxide/oxygen mixture (50% N_2_O/50% O_2_). Subsequently, a laryngeal mask was inserted. RA was performed using one of the described techniques under general anesthesia. To maintain anesthesia, sevoflurane at an end-tidal concentration from 0.8 to 1 MAC was used, along with an air/oxygen mixture of 0.5–1 L per minute and an inspiratory oxygen fraction of 0.3.

If the patient’s heart rate increased by more than 10% of baseline values during the operation, Remifentanil was intravenously administered at a dose of 1 µg/kg bodyweight. Systolic and diastolic blood pressure, as well as the heart rate, were recorded at three different time points: at the beginning of the surgical procedure (t0), during manipulation of the spermatic cord (t1), and near the end of anesthesia at the time of skin suturing (t2). The anesthesia was stopped after the surgery was completed.

The anesthesia induction time was recorded retrospectively using the operating theatre documentation.

### 2.5. Postoperative Management

**Figure 2:**
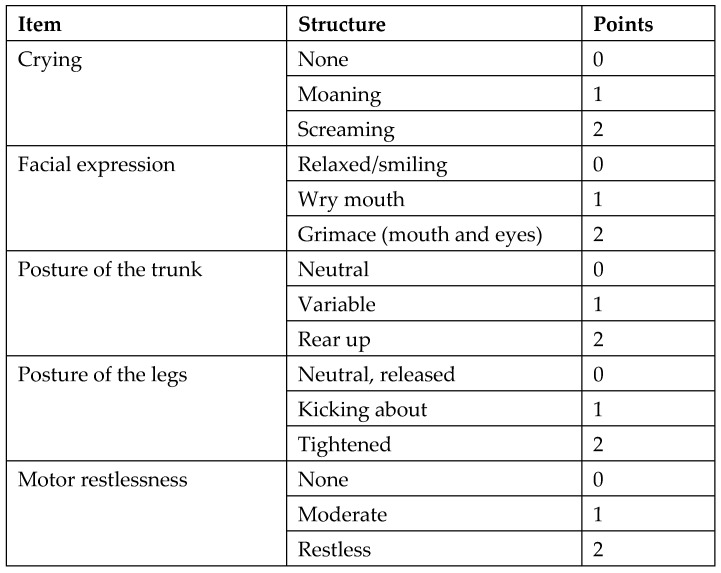
Children’s and Infants Postoperative Pain Scale (CHIPPS).

Following surgery, patients were transferred to the post-anesthetic care unit (PACU) where their hemodynamics and respiratory parameters were continuously monitored. Pain assessment was conducted every 15 min using the Children’s and Infants Postoperative Pain Scale (CHIPPS) in German (Figure 2. If the pain score exceeded 4, piritramide was intravenously administered at a dosage of 0.05–0.1 mg/kg body weight. Cardiac parameters were recorded before transferring patients to the general ward.

Patients were moved to the general ward once they achieved stable vital signs and a pain score below 4. Subsequent pain score assessments were conducted at four-hour intervals. If the pain score exceeded 4, metamizole was administered intravenously at a dosage of 15 mg/kg body weight (maximum dose of 60 mg/kg body weight) as a short infusion. Patients were discharged from the hospital on the morning of the first postoperative day.

### 2.6. Statistical Analysis

Statistical analyses were conducted using IBM SPSS Statistics 29. Baseline characteristics are presented as mean ± standard deviation or median (minimum–maximum), median (IQR), and as a number (percentage) for qualitative variables.

Normality was tested using frequency distribution (histogram). Unpaired or paired student’s t-tests were used for normally distributed ordinal data. The Mann–Whitney U test was used for non-normally distributed ordinal data. The study compared categorical data using either the χ^2^ test or Fisher’s exact tests, as appropriate. Additionally, a binary linear regression analysis was conducted to evaluate the relationship between multiple independent variables and a dependent variable. Independent risk factors were identified based on data that had a *p*-value < 0.05. The threshold for statistical significance was set at *p* < 0.05.

## 3. Results

During the 16-month study period, 71 boys underwent unilateral orchiopexy, with 34 of them obtaining an IINB and 37 receiving a CB. Table 1 presents detailed demographic data.

The comparative analysis revealed no statistically significant differences between the two groups in terms of weight, duration of surgery and anesthesia induction, and time spent in the PACU. However, a significant difference was observed in the age of the patients (*p* < 0.05). In a binary logistic regression, no significant risk was found regarding the need for analgesics based on age and weight in relation to the type of regional anesthesia. The blocks were tested against each other, using each block as a reference in turn (Table 2).

### 3.1. Intraoperative and PACU

**Figure 3:**
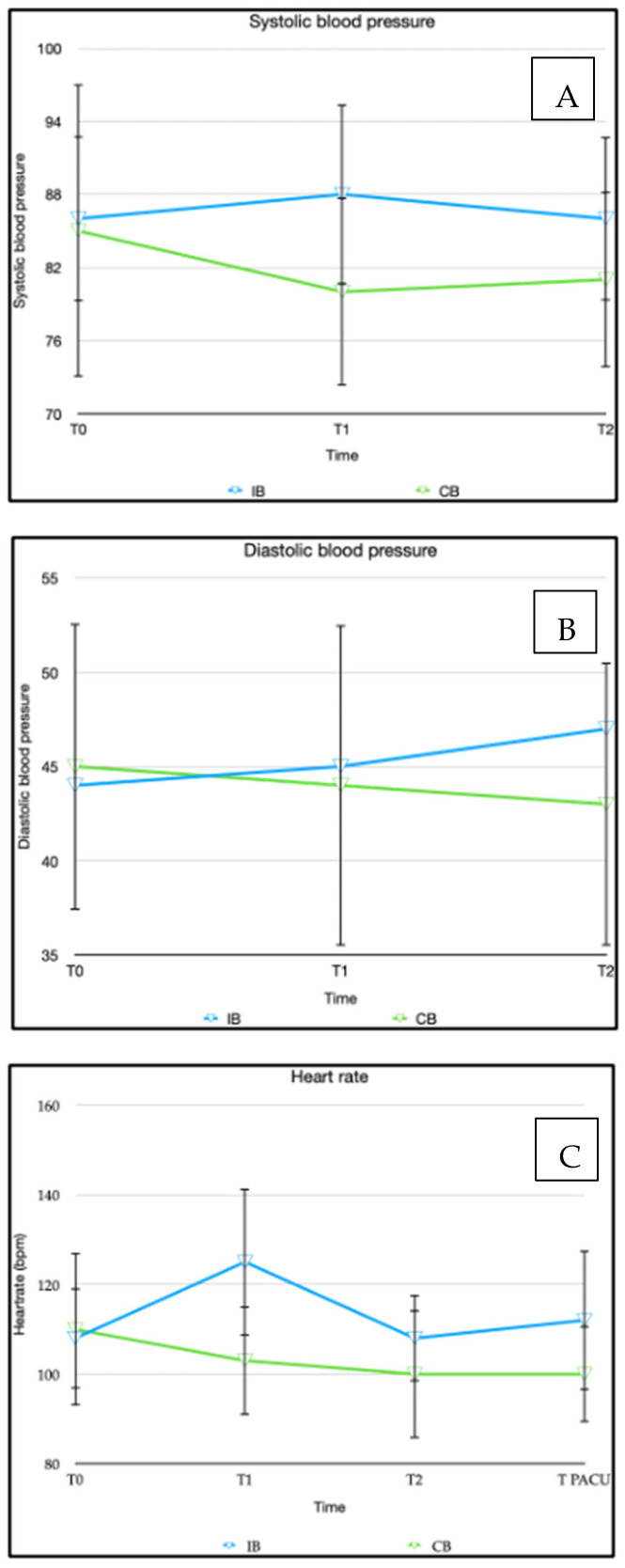
(**A**) Mean arterial systolic blood pressure; (**B**) mean arterial diastolic blood pressure; (**C**) heart rate values in both groups.

At the initial measurement point (T0), the baseline systolic and diastolic blood pressure, as well as the heart rate, were similar in both groups. However, the IINB group showed a significant increase in systolic blood pressure (<0.001) and a significant increase in heart rate (<0.001) when compared to the baseline values (Figure 3). 

In the CB group, none of the patients showed an increase in heart rate during the operation. However, in the IINB group, 26 out of 34 patients (76.5%) experienced a heart rate increase of more than 10% (*p* < 0.001). As a result, the administration of Remifentanil was significantly higher in the IINB group (*p* < 0.001). More than half of the patients in this group (53.8%) experienced multiple heart rate increases and required additional doses of Remifentanil.

In the PACU, 8 out of 37 patients (21.6%) from the CB group and 18 out of 34 patients (52.9%) from the IINB group required the administration of piritramide. The results demonstrated no statistically significant difference in terms of the level of the CHIPPS score (*p* = 0.533) or in the timing of piritramide administration (*p* = 0.106). Detailed data are presented in Table 3.

### 3.2. General Ward

For improved comparability of analgesic requirements on the ward, patients were stratified based on whether they received piritramide in the PACU. Piritramide, with a duration of action lasting 5–8 h, was treated as a distinct variable owing to its potential influence on subsequent analgesic administration.

Patients who did not receive piritramide in the PACU did not exhibit a significantly higher incidence of requiring metamizole on the ward (*p* = 0.070) compared to those who had received piritramide earlier.

Among the 29 patients in the CB group who did not receive piritramide in the PACU, 25 individuals (86.2%) subsequently underwent metamizole administration. In contrast, within the IINB group comprising 16 patients, 10 individuals (62.5%) received metamizole on the ward. However, no significant differences were observed between the two groups in terms of the onset of pain symptoms (*p* = 0.619), the level of CHIPPS score (*p* = 0.439), or the 24 h total dose of metamizole (*p* = 0.986).

Furthermore, among the eight patients in the CB group who received piritramide in the PACU, five (62.5%) required additional metamizole on the ward. In the IINB group, where 18 patients received piritramide in the PACU, 10 (55.6%) required metamizole on the ward. A significant difference in the onset of pain symptoms was demonstrated (*p* = 0.030), whereas no significant differences were observed in the level of the CHIPPS score (*p* = 0.513) and the 24 h total dose (*p* = 0.310). Further detailed data are shown in Table 4.

Regarding the occurrence of postoperative vomiting (PONV), the results demonstrate that in the CB group, five patients (13.5%) suffered from PONV, and in the IINB group, nine (26.5%) patients suffered. No significant difference was found (*p* = 0.235).

All patients were able to be discharged on the morning of the following day without any complications. None of the patients exhibited urinary retention or prolonged motor blockades during the monitoring phase.

Five patients (13.5%) in the CB group and two patients (5.9%) in the IINB group demonstrated no need for analgesics; however, no statistically significant difference was observed between the groups (*p* = 0.299). Consequently, seven patients (9.9%) within the entire study population did not necessitate analgesic administration over the entire 24 h duration of their hospital stay.

## 4. Discussion

### 4.1. Main Findings

In our prospective study comparing the efficacy of caudal and inguinal blocks in the treatment of intraoperative and postoperative pain during orchidopexy, we demonstrated that caudal block is the superior procedure both intraoperatively and immediately postoperatively. Although the differences between the groups were not significant, there was a slightly higher metamizole consumption pattern in the CB group compared to the IINB group in the 24 h postoperative phase. A slightly lower incidence of postoperative nausea and vomiting (PONV) was observed in patients undergoing caudal anesthesia, although this was not statistically significant. No significant complications were observed in either group.

### 4.2. Findings in the Context of Existing Evidence

The provision of adequate intra- and postoperative analgesia is a critical factor determining the success of any surgical procedure. It is evident that the incidence of pain, nausea, and vomiting following orchidopexy is higher than that observed after other inguinal procedures [12]. This is attributed to the manipulation of the spermatic cord during the procedure, which is a source of considerable discomfort for the patient undergoing this operation [6]. Therefore, it is of paramount importance to ensure that adequate pain management is provided especially in an outpatient setting, as the most common reasons for unplanned hospital admission is pain [13].

A number of studies have demonstrated the safety and efficacy of both regional anesthesia techniques as methods for postoperative analgesia [5,6,14,15]. However, the literature on intraoperative efficacy is still relatively sparse. In order to obtain a more objective assessment of the pain stimulus, Somri et al. examined the effect of CB and IINB by measuring intraoperative and postoperative catecholamine plasma levels. They found that catecholamine levels were reduced after the application of regional anesthesia in both procedures. Conversely, however, significantly higher catecholamine levels were observed in the IINB group at the conclusion of the operation and in the recovery room [4].

Our study did not include a measurement of catecholamine levels. However, during the intraoperative period, the IINB group exhibited a significant increase in systolic blood pressure and heart rate, especially at T1, which corresponded to the traction on the spermatic cord. Therefore, the administration of Remifentanil was significantly higher in the IINB group. This leads to the conclusion that the CB block appears to be superior to the IINB block in terms of intraoperative efficacy.

In recent years, various meta-analyses have been conducted to evaluate the postoperative effectiveness of caudal block with local regional anesthesia in pediatric procedures in the groin region. Depending on the meta-analysis, the time to administration of rescue analgesia, the number of patients requiring rescue analgesia, and the postoperative pain score were defined as outcome parameters [16,17,18,19].

In a recently published meta-analysis, Hung et al. found that in orchidopexy, CB was the only regional analgesia that prolonged the time to first rescue analgesia. In contrast, IINB had a relatively small analgesic effect [18]. A similar conclusion was reached by Shanthanna et al., who conducted a meta-analysis comparing caudal blocks with non-caudal blocks in inguinal procedures. The outcomes determined were efficacy based on analgesic requirement at 4 h and at 4–24 h. It was found that caudal anesthesia was more effective than local regional procedures, especially in the later period [19].

In contrast, Desai et al. found no difference between IINB and CB in terms of the 0–2 h postoperative pain score and the need for in-hospital rescue analgesia in their meta-analysis [16].

In a meta-analysis from 2013, which included a total of 13 studies from monocenters with small case numbers, Baird et al. also found no significant differences in the pain score one-hour postoperatively. Astonishingly, based on this balance, they postulated that in patients without additional indications for pain management, CB could be omitted for lower-risk and less time-consuming maneuvers. However, clinical adverse effects were not investigated in this meta-analysis [17].

In terms of clinical side effects, Shanthanna et al. found a higher risk of motor blockages and urinary retention [19]. Desai and colleagues also reached a similar conclusion regarding urinary retention, although this is attributed to a single included publication by Markham and colleagues [5,16]. In his study, Markham used isobaric bupivacain 0.5% for caudal anesthesia, which, in addition to its high concentration, has a higher tendency for motor blockade than the ropivacaine used in our study [5].

In our study, the CB block was found to be neither more time-consuming than the IINB block nor to result in any significant adverse effects, such as urinary retention or motor blockade.

Another crucial aspect, especially in outpatient surgery, is the potential for delayed discharge or unplanned hospital admission due to the occurrence of postoperative nausea and vomiting (PONV), post-discharge nausea and vomiting, or opioid-induced nausea and vomiting. It is widely acknowledged that multimodal pain management, including perioperative regional and opioid-sparing analgesia, is an effective strategy for preventing nausea and vomiting [13]. However, to the best of our knowledge, regarding groin surgery, this has not yet been a focus of the existing literature. In our study, we observed a significant reduction in the need for opioids in the CB group compared to the IINB group. Additionally, the incidence of PONV was lower in the CB group, although not to a statistically significant degree.

The available meta-analyses provide a clear illustration of the current inconsistency of the data situation. It is our conviction that the data of our study will serve to enhance the efficacy of pain management. Nevertheless, it should be noted that this study is not without limitations. Despite the prospective design, this study is subject to certain limitations. For instance, no randomization was conducted, which resulted in a difference in age between the two groups. However, the influence of age and height was ruled out using a binary logistic regression analysis. The additional use of clonidine in the context of CB might have further influenced the duration of analgesia. One of the objectives of this study was to compare the commonly used procedures in clinical practice to potentially derive recommendations. Another limitation is the assessment of pain intensity using the CHIPPS score, despite this being a validated instrument for assessment. In addition to the age-related limitations in the ability to adequately verbalize pain, other factors such as the unfamiliar environment, hunger, thirst, and limited freedom of movement influence one’s ability to assess pain.

### 4.3. Implications for Clinical Practice

Both CB and IINB demonstrate safety and reliability in providing sufficient regional analgesia for orchidopexy. Intraoperatively and during early recovery, CB exhibits superiority, especially in conjunction with supplementary opioid administration, potentially mitigating postoperative nausea and vomiting (PONV). Conversely, IINB may exhibit slight superiority in the late recovery phase. However, it is recognized that orchidopexy constitutes a procedure associated with considerable pain, often necessitating additional analgesia with non-steroidal anti-inflammatory drugs (NSAIDs) in the later postoperative phase. These considerations are particularly pertinent in the outpatient setting.

## 5. Conclusions

Our study has demonstrated that both the CB and IINB procedures represent safe and feasible methods. Despite the established status of both regional anesthesia techniques and the frequency of orchidopexy procedures, significant ambiguity and inconsistency persist in this domain. Therefore, a multicenter, randomized, prospective study would be desirable to gain further insights and establish clear guidelines.

## Figures and Tables

**Table 1:** Demographic data.

	CB Group (*n* = 37)	IINB Group (*n* = 34)	*p*-Value
Age (month) Median (range)	13 (10–55)	21 (10–50)	0.014
Weight (kg) Median (range)	11 (8–22)	12.3 (7.5–18)	0.177
Duration of surgery (min) Mean ± SD	44.1 ± 13.6	43.7 ± 12.1	0.963
PACU * time (min) Median (IQR)	105 (90–130)	90 (73.8–120)	0.109
Duration of anesthesia induction (min) Mean ± SD	14.9 ± 6.7	14.2 ± 4.8	0.764

* PACU: post-anesthetic care unit, IQR: interquartile range; significance (*p* < 0.05) is highlighted in bold.

**Table 2:** Analysis of pain medication requirements.

	*p*	OR	95% CI
CB block	0.003	7.281	1.954–27.128
Age	0.850	1.008	0.925–1.099
Weight	0.645	1.095	0.745–1.609
IIHB block	0.003	0.137	0.037–0.512
Age	0.850	1.008	0.925–1.099
Weight	0.645	1.095	0.745–1.609

**Table 3:** Pain medication: administration, timing, and level of pain in PACU.

	CB Group (*n* = 37)	IINB Group (*n* = 34)	*p*-Value *
Number of pat. requiring i.v. piritramide in PACU	8 (21.6%)	18 (52.9%)	0.008
Median level of CHIPPS score in PACU (range)	5 (4–8)	5 (4–10)	0.533
Mean time of first CHIPPS score in PACU (range)	60 (15–180)	45 (15–120)	0.106

* Significance (*p* < 0.05) is highlighted in bold.

**Table 4:** Pain medication: administration, timing, and level of pain on ward.

	CB Group	IINB Group	*p*-Value
Total number of pat. requiring i.v. metamizole on ward	30/37 (81.1%)	20/34(58.8%)	0.067
With piritramide	5/8 (62.5%)	10/18 (55.6%)	1.000
Without piritramide	25/29 (86.2%)	10/16 (62.5%)	0.070
Total amount (median (IQR)) of metamizole (mg)/24 h	150 (115–212.5)	187.5 (105–335)	0.517
With piritramide	150 (120–150)	187.5 (123.75–391.25)	0.310
Without piritramide	150 (110–260)	185 (100–305)	0.986
Median level of CHIPPS score on ward (range)	5 (2–10)	5 (4–8)	0.577
With piritramide	4 (4–7)	5 (4–8)	0.513
Without piritramide	5 (2–10)	4.5 (4–6)	0.439
Mean time (min) of first CHIPPS score on ward (range)	300 (60–840)	540 (60–840)	0.050
With piritramide	400 (240–435)	577.5 (60–840)	0.030 *
Without piritramide	300 (60–840)	300 (120–840)	0.619

* Significance is shown in bold.

## Data Availability

The data used to support the findings of this study are available from the corresponding author upon request.
